# Exome Sequencing Data Analysis and a Case-Control Study in Mexican Population Reveals Lipid Trait Associations of New and Known Genetic Variants in Dyslipidemia-Associated Loci

**DOI:** 10.3389/fgene.2022.807381

**Published:** 2022-05-20

**Authors:** Pedro A. Jurado-Camacho, Miguel A. Cid-Soto, Francisco Barajas-Olmos, Humberto García-Ortíz, Paulina Baca-Peynado, Angélica Martínez-Hernández, Federico Centeno-Cruz, Cecilia Contreras-Cubas, María Elena González-Villalpando, Yolanda Saldaña-Álvarez, Guadalupe Salas-Martinez, Elvia C. Mendoza-Caamal, Clicerio González-Villalpando, Emilio J. Córdova, Lorena Orozco

**Affiliations:** ^1^ Immunogenomics and Metabolic Diseases Laboratory, National Institute of Genomic Medicine, Mexico City, Mexico; ^2^ Posgraduate in Biomedical Sciences, National Autonomous University of Mexico, Mexico City, Mexico; ^3^ Centro de Estudios en Diabetes, Unidad de Investigación en Diabetes y Riesgo Cardiovascular, Centro de Investigación en Salud Poblacional, Instituto Nacional de Salud Pública, Mexico City, Mexico; ^4^ Clinical Area, National Institute of Genomic Medicine, Mexico City, Mexico; ^5^ Oncogenomics Consortium Laboratory, National Institute of Genomic Medicine, Mexico City, Mexico

**Keywords:** association study, genetic variants, dyslipidemia, exome analysis, mexican population

## Abstract

**Background:** Plasma lipid levels are a major risk factor for cardiovascular diseases. Although international efforts have identified a group of loci associated with the risk of dyslipidemia, Latin American populations have been underrepresented in these studies.

**Objective:** To know the genetic variation occurring in lipid-related loci in the Mexican population and its association with dyslipidemia.

**Methods:** We searched for single-nucleotide variants in 177 lipid candidate genes using previously published exome sequencing data from 2838 Mexican individuals belonging to three different cohorts. With the extracted variants, we performed a case-control study. Logistic regression and quantitative trait analyses were implemented in PLINK software. We used an LD pruning using a 50-kb sliding window size, a 5-kb window step size and a r^2^ threshold of 0.1.

**Results:** Among the 34251 biallelic variants identified in our sample population, 33% showed low frequency. For case-control study, we selected 2521 variants based on a minor allele frequency ≥1% in all datasets. We found 19 variants in 9 genes significantly associated with at least one lipid trait, with the most significant associations found in the *APOA1/C3/A4/A5-ZPR1-BUD13* gene cluster on chromosome 11. Notably, all 11 variants associated with hypertriglyceridemia were within this cluster; whereas variants associated with hypercholesterolemia were located at chromosome 2 and 19, and for low high density lipoprotein cholesterol were in chromosomes 9, 11, and 19. No significant associated variants were found for low density lipoprotein. We found several novel variants associated with different lipemic traits: rs3825041 in *BUD13* with hypertriglyceridemia, rs7252453 in *CILP2* with decreased risk to hypercholesterolemia and rs11076176 in *CETP* with increased risk to low high density lipoprotein cholesterol.

**Conclusions:** We identified novel variants in lipid-regulation candidate genes in the Mexican population, an underrepresented population in genomic studies, demonstrating the necessity of more genomic studies on multi-ethnic populations to gain a deeper understanding of the genetic structure of the lipemic traits.

## Introduction

Currently, metabolic diseases have become one of the most challenging health concerns worldwide; they account for nearly 45% of all deaths worldwide (Forouzanfar et al., 2016). Prevention and management of these diseases are complicated, due to their long latency periods, numerous risk factors, and the presence of co-morbidities (Sevick et al., 2007). Among the known metabolic diseases, dyslipidemias comprise a well-established risk factor for cardiovascular diseases, which are the leading cause of death worldwide (Libby, 2002).

Although factors like diet and lifestyle (Wietlisbach et al., 1997) are recognized as important determinants in the clinical development of dyslipidemias, these diseases have also a strong genetic component (Gao et al., 2018). For instance, lipid disorders are highly prevalent in populations with Amerindian ancestry (up to 85.9%), compared with the prevalence among individuals from other ancestries (e.g., Caucasian 31.2% and African 41.1%) (Aguilar-Salinas et al., 2014; Noubiap et al., 2018). In addition, a large number of genome-wide association studies (GWAS) performed in populations of different ancestries have described more than 175 loci associated with dysregulated levels of plasma lipids. However, the largest body of information generated to date has relied mostly on evidence from Caucasian and Asian cohorts, with very few studies analyzing Latin American populations (Teslovich et al., 2010; Asselbergs et al., 2012; Willer et al., 2013; Wu et al., 2013; Surakka et al., 2015).

Recent findings have demonstrated that genetic factors associated with metabolic traits, including dyslipidemias, exhibited significant heterogeneity in allele frequency and in variant effects across groups with different ancestries (Klarin et al., 2018; Martagón et al., 2018). A study analyzing populations from multiple ethnicities has found important differences in the levels of association, allele frequencies, and haplotype distributions of several lipid loci (Wu et al., 2013). Several population-specific signals at these loci have been reported in non-European populations. For example, the association between the regulatory variant, rs12740374, in the *CELSR2/PSRC1/SORT1* locus, and low-density lipoprotein cholesterol (LDL-C) is higher in African-American individuals than in individuals of European descent (Buyske et al., 2012). Furthermore, the R230C variant of the *ABCA1* gene, which is associated with low levels of high-density lipoprotein cholesterol (HDL-C), is private to individuals with Amerindian ancestry (Acuña-Alonzo et al., 2010). Thus, the distribution of variants located in lipid-related genes might vary between populations of different ethnicities.

The modern Mexican population is mainly composed of Mestizo individuals, who are the result of the recent admixture of original Amerindians, Europeans (mainly Spaniards) and, to a lesser extent, sub-Saharan Africans (Moreno-Estrada et al., 2014). The recent and complex admixture in the Mexican Mestizo population might have produced a high level of genetic heterogeneity in variants in lipid-related loci. Therefore, this study aimed to determine the frequency distribution of variants located at genes related to lipid traits in Mexican individuals, followed by an association testing and the identification of potential variants for lipemic traits, using previously published exome sequencing data (Estrada et al., 2014; García-Ortíz et al., 2021).

## Material and Methods

### Study Populations

This study included 2838 Mexican individuals belonging to the Mestizo cohorts Diabetes in Mexico Study (DMS, *n* = 968) and Mexico City Diabetes Study (MCDS, *n* = 796), published previously as part of the Slim Initiative in Genomic Medicine for the Americas (SIGMA) Type 2 Diabetes Consortium (Estrada et al., 2014), as well as to the indigenous cohort Metabolic Analysis in an Indigenous Sample (MAIS, *n* = 1074) (García-Ortíz et al., 2021). The sample design was previously described in Estrada et al. and García-Ortiz et al. (Estrada et al., 2014; García-Ortíz et al., 2021). The MAIS sample belongs to 71 indigenous communities representing 60 ethnic groups from 10 linguistic families. All participants were unrelated volunteers and provided signed informed consent. This investigation was approved by the local ethics and research committees from the National Institute of Genomic Medicine and was conducted according to the principles of the Declaration of Helsinki. The genetic structure of DMS and MCDS population was previously described, with a mean (SD) proportion of Amerindian ancestry of 66 ± 17%, whereas the proportion of Amerindian ancestry in the MAIS cohort was of 93.2 ± 8.7% (Estrada et al., 2014; García-Ortíz et al., 2021).

### Lipid Measurements in Plasma

Levels of triglycerides (TG), total cholesterol (TC), and HDL-C were measured from blood samples collected after overnight fasting, using a Synchron CX5 Analyzer System (Beckman Coulter Fullerton, CA, United States). LDL-C values were calculated with the Friedewald formula, excluding those samples with TG > 400 mg/dl (Warnick et al., 1990).

Each lipid disorder was diagnosed according to the American Heart Association and National Heart, Lung, and Blood Institute guidelines (AHA/NHLBI; http://www.nhlbi.nih.gov). An individual was diagnosed with a lipid disorder when serum levels showed any of the following: TG ≥ 150 mg/dl (hypertriglyceridemia; HTG), TC ≥ 200 mg/dl (hypercholesterolemia; HTC), LDL-C ≥130 mg/dl (elevated LDL), or HDL-C ≤50 mg/dl in females or ≤40 mg/dl in males (low HDL-C). Individuals with desirable lipid values were assigned as controls. Data on lipid-lowering medications were available for over 80% of the participants and adjustment was done for TG and TC binary phenotypes.

### Dataset Building and Single-Nucleotide Variant Annotations

First, we analyzed the Variant Call Format (VCF) files previously obtained from all participants. Quality controls for whole-exome sequencing (e.g., read depth, mean coverage, and missing rate) were described previously (Estrada et al., 2014). From these files, we extracted all the biallelic variants (mapped in the Genome Reference Consortium Human genome build 37) of 177 candidate genes for any lipid trait (Supplementary Table S1). These genes were selected from: 1) the Global Lipid Genetics Consortium (*n* = 165) (Willer et al., 2013), 2) meta-analysis studies (*n* = 10) (Teslovich et al., 2010; Weissglas-Volkov and Pajukanta, 2010), and 3) re-sequencing and clinical exome studies (*n* = 2) (Estrada et al., 2014; Williams-Amy et al., 2014). We annotated the variants with the ENSEMBL Variant Effect Predictor (https://www.ensembl.org/info/docs/tools/vep/index.html; version 87) (McLaren et al., 2016). Finally, we constructed PLINK files that comprised clinical-demographic and genotyping data for the association analyses, which employed VCF tools (v0.1.12b) (Danecek et al., 2011).

### Statistical Analysis

A case-control study was conducted in each cohort to identify low-frequency SNVs [minor allele frequency (MAF) = 0.01–0.05] and common SNVs (MAF >0.05) associated with lipid traits. To analyze associations between lipid components and alleles we estimated the odds ratio (OR) using logistic regression. We performed quantitative trait analyses with linear regression (Beta value). Both methods were performed using an additive model, adjusting by age, sex and the first 10 eigenvectors from the principal component analysis as covariates. All analyses were performed using PLINK v.1.9 software (Purcell et al., 2007).

Next, we performed a fixed effects model meta-analysis for each trait using a weighted inverse variance model in the software package METAL (Willer et al., 2010). Also, genomic control was applied to each study within METAL by adjusting for the genomic inflation factor prior to meta-analysis, to correct for possible residual population stratification.

Significant threshold was determined following the approach by Kanai et al. (Kanai et al., 2016). According to this approach an LD pruning was done with PLINK v.1.9 using a 50-kb sliding window size, a 5-kb window step size and a r^2^ threshold of 0.1. According with this, a total of 780 independent SNVs were identified giving a significant threshold of 6.4 × 10^–5^.

Significant threshold was determined following the approach by Kanai et al. (Kanai et al., 2016). According to this approach, high LD SNVs in 177 candidate lipids genes were filtered based on LD pruning performed in PLINK v.1.9 using a 50-kb sliding window size, a 5-kb window step size and a r^2^ threshold of 0.1. According with this, a total of 780 independent SNVs were identified that were used to establishing the genome-wide significant threshold *via* a straight-forward Bonferroni correction *p* < (6.4 × 10^–5^).

## Results

### Study Participants

Our study population comprised 2838 individuals belonging to three different cohorts, the Diabetes in Mexico Study (DMS), the Mexico City Diabetes Study (MCDS) and the indigenous cohort Metabolic Analysis in an Indigenous Sample (MAIS) (Table 1). The DMS sample was composed of 968 individuals: 681 females (70.3%) and 287 males (29.7%). The mean age of the participants was 54.1 ± 9.8 years and the mean body mass index BMI was 28.4 ± 4.9 kg/m^2^. According to the AHA/NHLBI cutoff points, the lipid trait with the highest prevalence in this sample was low HDL-C (70.8%), followed by HTC (65.1%), HTG (54.9%), and high LDL-C (37%). The MCDS sample consisted of 796 individuals: 482 females (60.6%) and 314 males (39.4%) with a mean age of 62.9 ± 7.6 years and a mean BMI of 29.5 ± 4.9 kg/m^2^. The most frequent lipid alteration in this population was low HDL-C (82.8%), followed by HTG (58.8%), HTC (44.1%), and high LDL-C (40.0%). The MAIS sample was composed of 1074 individuals: 679 females (63.2%) and 395 males (36.8%) with a mean age of 58.6 ± 12.1 years and a mean BMI of 27.5 ± 5.0 kg/m^2^. Similar to the two previous cohorts, low HDL-C (75.2%) was the most common lipid trait, followed by HTG (66.8%), HTC (29.7%) and high LDL-C (14.9%) (Table 1).

**TABLE 1:** Clinical and demographic characteristics of the studied cohort.

Characteristic		DMS	MCDS	MAIS
		*n* = 968	*n* = 796	*n* = 1074
Women/Men (%)		70.3/29.7	60.6/39.4	63.2/36.8
Age (Years)		54.1 ± 9.8	62.9 ± 7.6	58.6 ± 12.1
BMI (kg/m^2^)		28.4 ± 4.9	29.5 ± 4.9	27.5 ± 5.0
FG (mg/dl)		125.2 ± 61.4	114.3 ± 54.2	114.2 ± 63.9
HTG (%)		54.9	58.8	66.8
Mean TG (mg/dl)		203.7 ± 129.3	176.3 ± 96.3	207.5 ± 119.5
HTC (%)		65.1	44.1	29.7
Mean TC (mg/dl)		201.1 ± 43.8	195.9 ± 36.5	182.8 ± 38.3
Low HDL-C (%)		70.8	82.8	75.2
Mean HDL-C (mg/dl)	Women	39.7 ± 12.1	31.5 ± 8.5	40.6 ± 12.6
	Men	37.4 ± 13.0	28.6 ± 13.4	38.7 ± 12.6
High LDL-C (%)		37	40.0	14.9
Mean ^1^LDL-C (mg/dl)		120.7 ± 35.3	131.5 ± 33.6	103.7 ± 29.5

Data are presented as the mean ± SD, or the percentage, as indicated. BMI, body mass index; FG, fasting glucose; HTG, hypertriglyceridemia; TG, triglycerides; HTC, hypercholesterolemia; TC, total cholesterol; HDL-C, high density lipoprotein; LDL-C, Low density lipoprotein. ^1^LDL-C values were calculated with the Friedewald equation.

### Analysis of the Variation in Candidate Dyslipidemia-Related Genes

**FIGURE 1:**
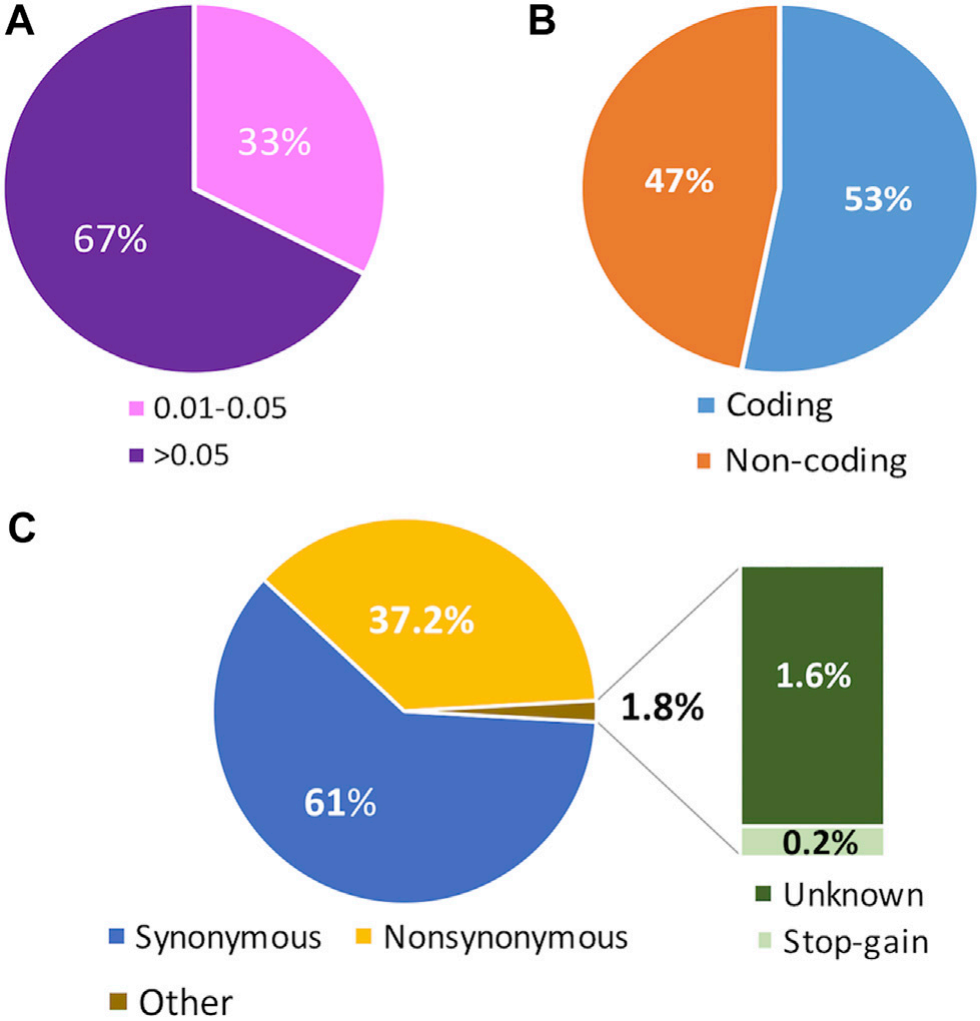
Features of the analyzed gene variant set. **(A)** Cumulative variant distribution according to the minor allele frequency (MAF). Rare variants include Singletons, Doubletons, and variants with MAFs <0.01. Low Frequency variants include variants with MAFs = 0.01–0.05. Common variants are those with MAFs >0.05. **(B)** Distribution of coding and non-coding annotations, according to the Variant Effect Predictor tool (VEP) in the entire set of variants.

After analyzing the exome data from the three analyzed cohorts, we found a total of 34251 biallelic variants with a MAF above 1% within the 177 genes previously related to dyslipidemia. Among these variants, 33% were detected at low frequency (MAF = 0.01–0.05), and 67% were common (MAF >0.05) (Figure 1A). According to their position in the gene, 53% of the variants were in coding regions, and the remaining 47% were found in non-coding regions (Figure 1B). According to the predicted annotation obtained with the Variant Effect Predictor web tool (https://grch37.ensembl.org/Tools/VEP) 61% of the variants found in the coding regions were synonymous, 37.2% were non-synonymous, 0.2% were stop-gain and 1.6% were unknown (Figure 1C).

### Low-Frequency and Common Variant Association Study

**FIGURE 2:**
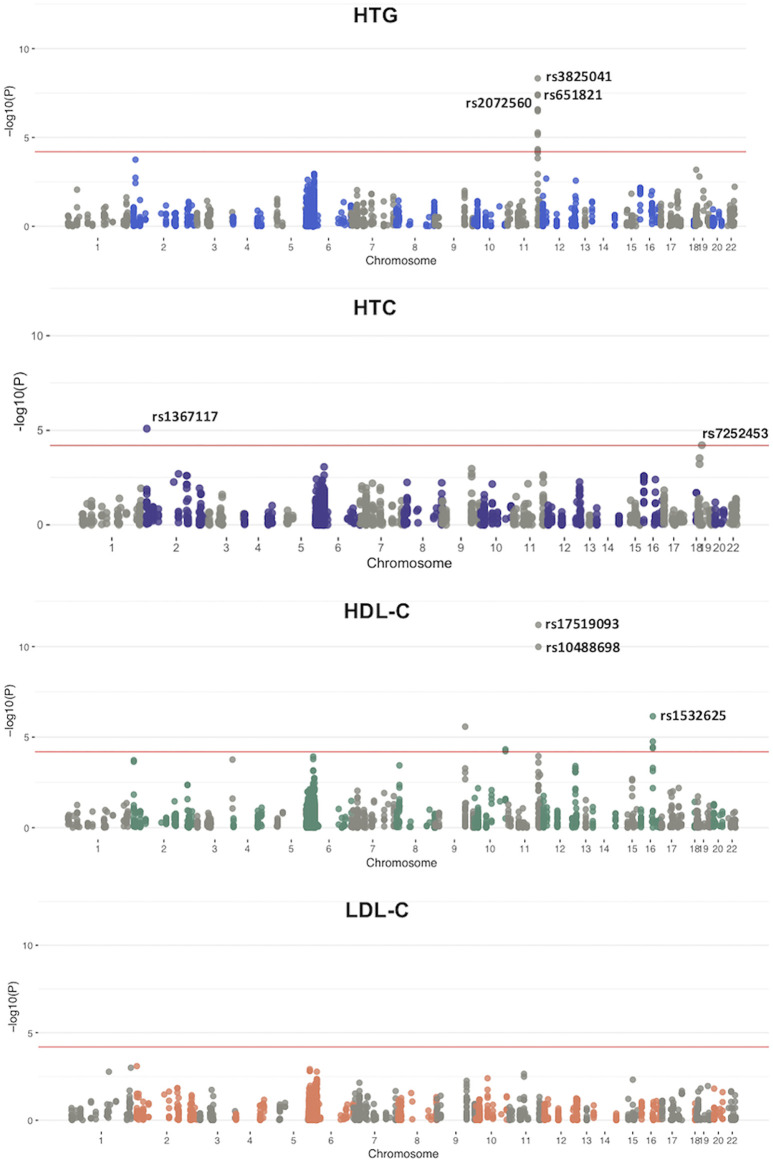
Manhattan plots from a meta-analysis based on the genome-wide association analysis. Panels show the −log *p* value for SNVs for each lipemic traits. Top hits for each trait are indicated in the figure. Red line indicates the significant threshold line: *p* = 6.4 × 10^−5^.

For the case-control study we used a meta-analysis approach to evaluate low-frequency and common variants pruned for LD (MAF >0.01; *n* = 780). We explored associations between individual variants and each different lipid phenotype with binary logistic regression. Quantile-quantile (QQ) plots for each analyzed phenotype showed a genomic inflation factor for HTG, HTC, low HDL-C and high LDL-C of λ = 1.0, 1.02, 1.03, and 0.91 respectively (Supplementary Figure S1). A total of 19 variants in 9 genes achieved a *p* < 6.4 × 10^–5^ or higher significant association with at least one of the analyzed lipid components (Figure 2). Remarkably, the 11 variants that were associated with HTG were found on chromosome 11, located in *BUD13*, *APOA5, APOC3*, *APOA1*, and *APOA4* genes (Supplementary Table S2). In the case of HTC, associated variants were found on chromosome 2 and 19, in *APOB* and *CILP2* genes; whereas in the case of low HDL-C, two of the associated variants were found on chromosome 11, in *BUD13* gene, four on chromosome 16 in *CETP* and one on chromosome 9 in *ABCA1* (Supplementary Table S2). We did not find any variant associated with high LDL-C.

**FIGURE 3:**
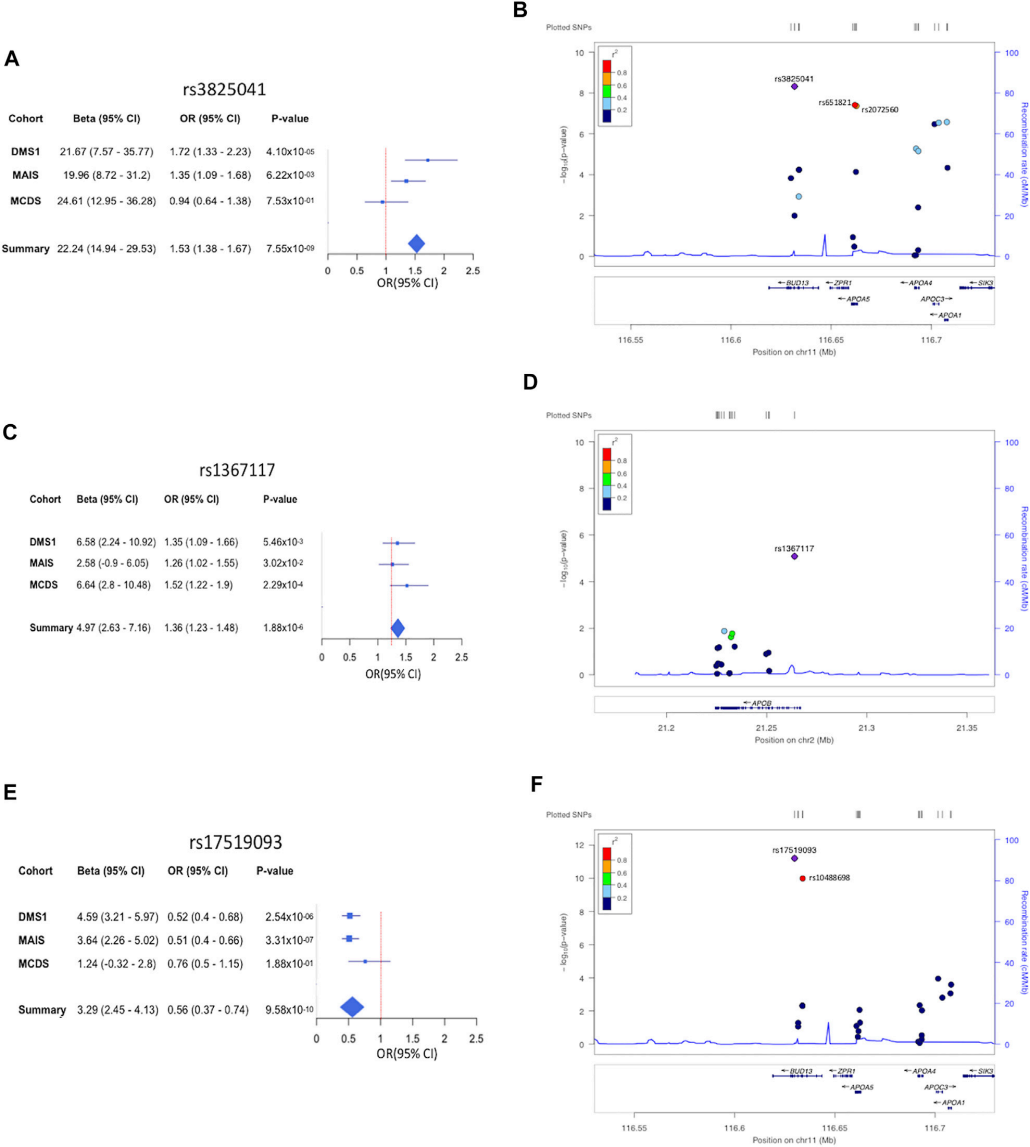
Forest and regional plots showing the top hits associated with lipemic traits. Forest plot showing odds ratio estimates and 95% confidence intervals (squared boxes) from the 3 cohorts DMS, MCDS, and MAIS, included in the study in HTG **(A)**, HTC **(C)**, and low HDL-C **(E)**. Odds ratios for the meta-analyses are represented with a diamond. Regional plot of associated SNVs in HTG **(B)**, HTC **(D)**, and low HDL-C **(F)**.

The 11 variants associated with HTG were all located in the *APOA1/C3/A4/A5-ZPR1-BUD13* gene cluster (Figure 2; Supplementary Table S2). The strongest signal for the variants associated with increased risk to HTG was detected for rs3825041 in *BUD13* (OR = 1.53, *p =* 7.55 × 10^−9^; β = 22.24 mg/dl) (Figure 3; Supplementary Table S2), followed by rs651821 (OR = 1.48, *p =* 5.70 × 10^−8^; β = 21.40 mg/dl) and rs2072560 in *APOA5* (OR = 1.48, *p =* 6.18 × 10^−8^; β = 21.65 mg/dl), rs2070665 in *APOA1* (OR = 1.41, *p =* 3.79 × 10^−7^; β = 17.71 mg/dl), rs5128 in *APOC3* (OR = 1.40, *p =* 4.22 × 10^−7^; β = 18.31 mg/dl), rs5104 (OR = 1.34, *p =* 6.1 × 10^−6^; β = 19.61 mg/dl) and rs5092 in *APOA4* (OR = 1.33, *p =* 8.07 × 10^−6^; β = 18.97 mg/dl) and rs11820589 in *BUD13* (OR = 1.35, *p =* 5.71 × 10^−5^; β = 21.92 mg/dl). In contrast, the variants rs4520 in *APOC3* (OR = 0.74, *p* = 2.96 × 10^−7^; β *=* −19.03 mg/dl) rs5070 in *APOA1* (OR = 0.78, *p* = 4.27 × 10^−5^; β *=* −15.75 mg/dl) and rs10488698 in *BUD13* (OR = 0.73, *p* = 5.80 × 10^−5^; β *=* −20.98 mg/dl) were associated with protection against HTG. All 11 variants were also significantly associated with TG levels in the quantitative trait analysis (Supplementary Figure S2; Supplementary Table S2).

Moreover, the variant rs1367117 in *APOB* (OR = 1.36, *p* = 1.88 × 10^−6^; β = 4.89) was significantly associated with HTC and with a significant increase in serum TC levels in the quantitative trait analysis (Figure 3; Supplementary Table S2). On the other hand, the variant rs7252453 in *CILP2* (OR = 0.73, *p* = 4.40 × 10^−5^; β = −5.17) was significantly associated with protection against HTC (Supplementary Figure S3; Supplementary Table S2). Regarding low HDL-C, the variants rs17519093 and rs10488698 in *BUD13* (OR = 0.56, *p* = 9.58 × 10^−10^, β = 3.29; OR = 0.58, *p* = 7.34 × 10^−9^; β = 3.12, respectively) and rs1532625 in *CETP* (OR = 0.72, *p* = 6.56 × 10^−6^; β = 2.21) were significantly associated with decreased risk in both the binary and the quantitative trait analysis (Figure 3; Supplementary Figure S4; Supplementary Table S2). Finally, the variants rs708272, rs11076176, and rs289714 in *CETP* (OR = 1.32, *p* = 1.75 × 10^−5^, β = −2.12; OR = 1.32, *p* = 3.64 × 10^−5^, β = −1.83; OR = 1.32, *p* = 4.03 × 10^−5^, β = −1.75, respectively) as well as rs9282541 in *ABCA1* (OR = 1.67, *p* = 3.34 × 10^−5^; β = −2.50) showed a significant association with increased risk to low HDL-C in both the binary and the quantitative trait analysis (Supplementary Figure S4; Supplementary Table S2).

## Discussion

The contribution of genetic variation to human diseases is widely recognized (Xue et al., 2012). In particular, loss-of-function or modifier variants are usually related to changes in the biological activity of the corresponding gene. Several genomic regions are currently recognized as drivers of dyslipidemias in populations from several ethnicities, although in some populations, such as those of Amerindian or African origin, genomic studies have been scarce. Furthermore, many of these loci have displayed highly different associations with these entities across different ancestries (Lek et al., 2016; Martin et al., 2017). Therefore, lipid-associated genes should be analyzed in all populations.

Here, by analyzing SNVs within 177 candidate genes, we found several associations with different types of dyslipidemias in Mexican Mestizos, an admixed population with strong Amerindian (51%) and European (46%) components (Norris et al., 2018).

In our population we were able to replicate several associations with different lipemic traits previously reported in other ethnicities. For example, variants in the gene cluster *APOA1/C3/A4/A5-ZPR1-BUD13*, such as the regulatory SNVs rs5128 in *APOC3*, and rs651821 in *APOA5*, as well as the missense SNV rs2072560 also within *APOA5*, and the intronic variant rs2070665 in *APOA1*, were all associated with HTG in our population, as they are in European, Asian, and African populations (Feng et al., 2016; Fu et al., 2015; Jasim et al., 2018; Ken-Dror et al., 2010; Song et al., 2015; Zhou et al., 2013). Likewise, the association of the missense SNV rs1367117 in *APOB* with HTC has also been reported in populations of European ancestry (Lu et al., 2010), whereas the association of the missense SNVs rs10488698 in *BUD13* and rs9282541 in *ABCA1* with low HDL-C has also been observed in Asian and Latino American populations, respectively (Zhang et al., 2017; Acuña-Alonso et al., 2010). We also observed novel lipemic trait–associated variants in *BUD13*, such as rs3825041 and rs17519093, both localized within introns, associated with HTG and high levels of HDL-C, respectively. Using public data contained in the Common Metabolic Diseases Knowledge Portal (https://t2d.hugeamp.org/), we were able to confirm the associations of rs3825041 and rs17519093 with different lipemic traits. Thus, one of the genomic regions most consistently associated with lipid traits in human populations of diverse ethnic origins is the cluster *APOA1/C3/A4/A5-ZPR1-BUD13* (Teslovich et al., 2010; Willer et al., 2013; Parra et al., 2017; Bai et al., 2019). Importantly, all 11 variants associated with HTG in our study, were in this cluster. Among them, the novel variant rs3825041 in *BUD13* showed the strongest association. Notably, this variant showed high LD with rs651821 (r^2^ = 0.84) and rs2072560 (r^2^ = 0.89) within *APOA5*, which have both been previously reported as associated with HTG in several populations of different ancestries, including in Mexicans (Ken-Dror et al., 2010; Kim et al., 2019). Taken together, these data provide more insights about the variants at the *APOA1/C3/A4/A5-ZPR1-BUD13* gene cluster as a relevant risk factor for dyslipidemias such as HTG and low HDL-C, and highlight the notion that these could be biomarkers for susceptibility to these traits.

Others novel association signals were observed with the synonymous SNV rs7252453 in *CILP2* and decreased risk to HTC and the intronic SNV rs11076176 in *CETP* and increased risk to low HDL-C serum levels. On *CETP* we also observed an association of the intronic variants rs708272 and rs289714 with high risk to low HDL-C, as well as the association of the intronic variant rs1532625 with protection to this dyslipidemia. These findings are in line with those reported previously in Mexican individuals (Vargas-Alarcon et al., 2018), in a meta-analysis involving six independent Hispanic cohorts (Gao et al., 2018) and in Chinese population (Guo et al., 2015).

In summary, despite our selecting candidate genes were previously associated with dyslipidemia in other populations, we were able to find additional variants showing the strongest associations with lipid traits in Mexican individuals. These differences support the notion that high allelic heterogeneity exists in lipid loci across populations. Remarkably, the new associations of variants in genes previously related to dyslipidemia, points out the importance of studying different ethnicities, since different associated variants within the same genes could be particular to one or another population ancestry. It is also worth to note that several of the risk variants previously associated with lipemic traits in different ethnic groups, including European, Asian and African populations were replicated in our study. Taken together, our results suggest that genetic architecture of dyslipidemias is partially share among different ethnic groups.

## Data Availability

The data presented in the study are deposited in the European Variation Archive repository (EVA https://www.ebi.ac.uk/eva/), accession number PRJEB52611.
