# Supplementary figures and images for: Exome Sequencing Data Analysis and a Case-Control Study in Mexican Population Reveals Lipid Trait Associations of New and Known Genetic Variants in Dyslipidemia-Associated Loci
Source: Front Genet. 2022 May 20;13:807381. doi: 10.3389/fgene.2022.807381 (PMC9164108; doi:10.3389/fgene.2022.807381)

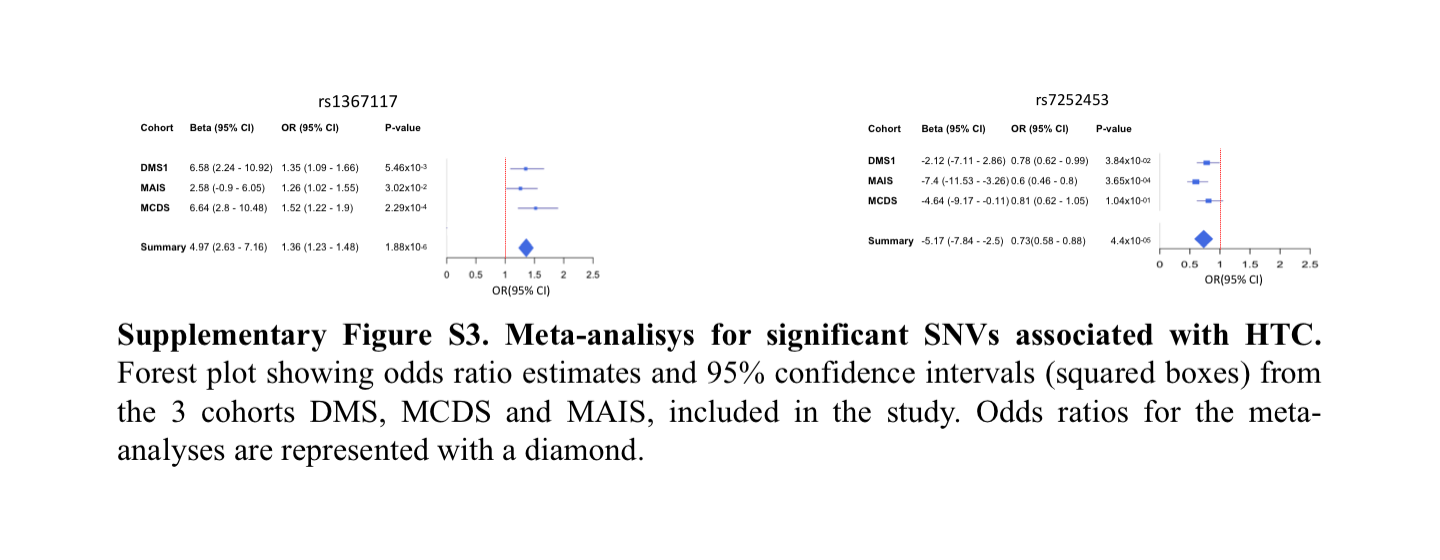

Supplement: Supplementary file 1 [file Image3.tiff]

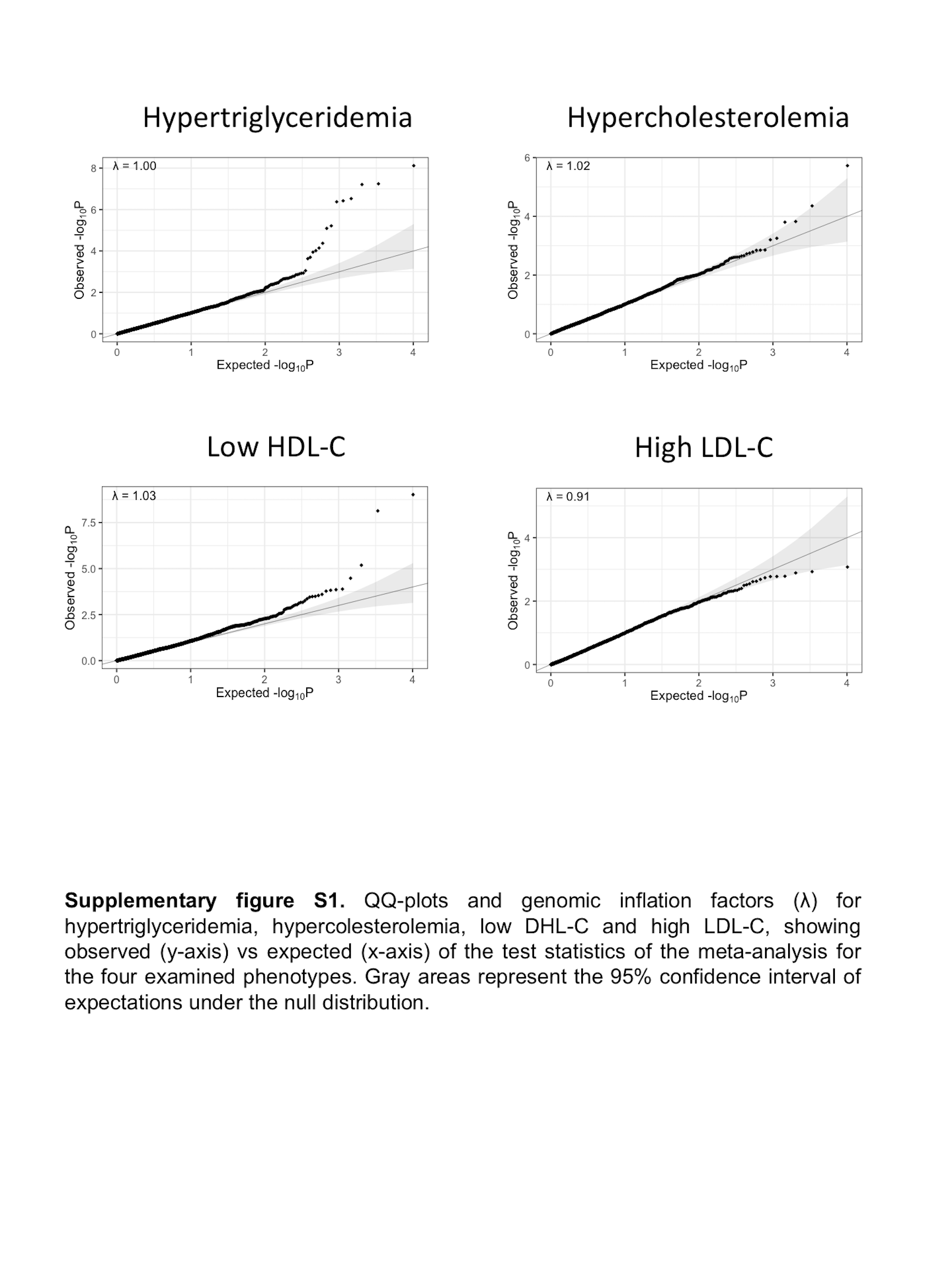

Supplement: Supplementary file 3 [file Image1.tiff]

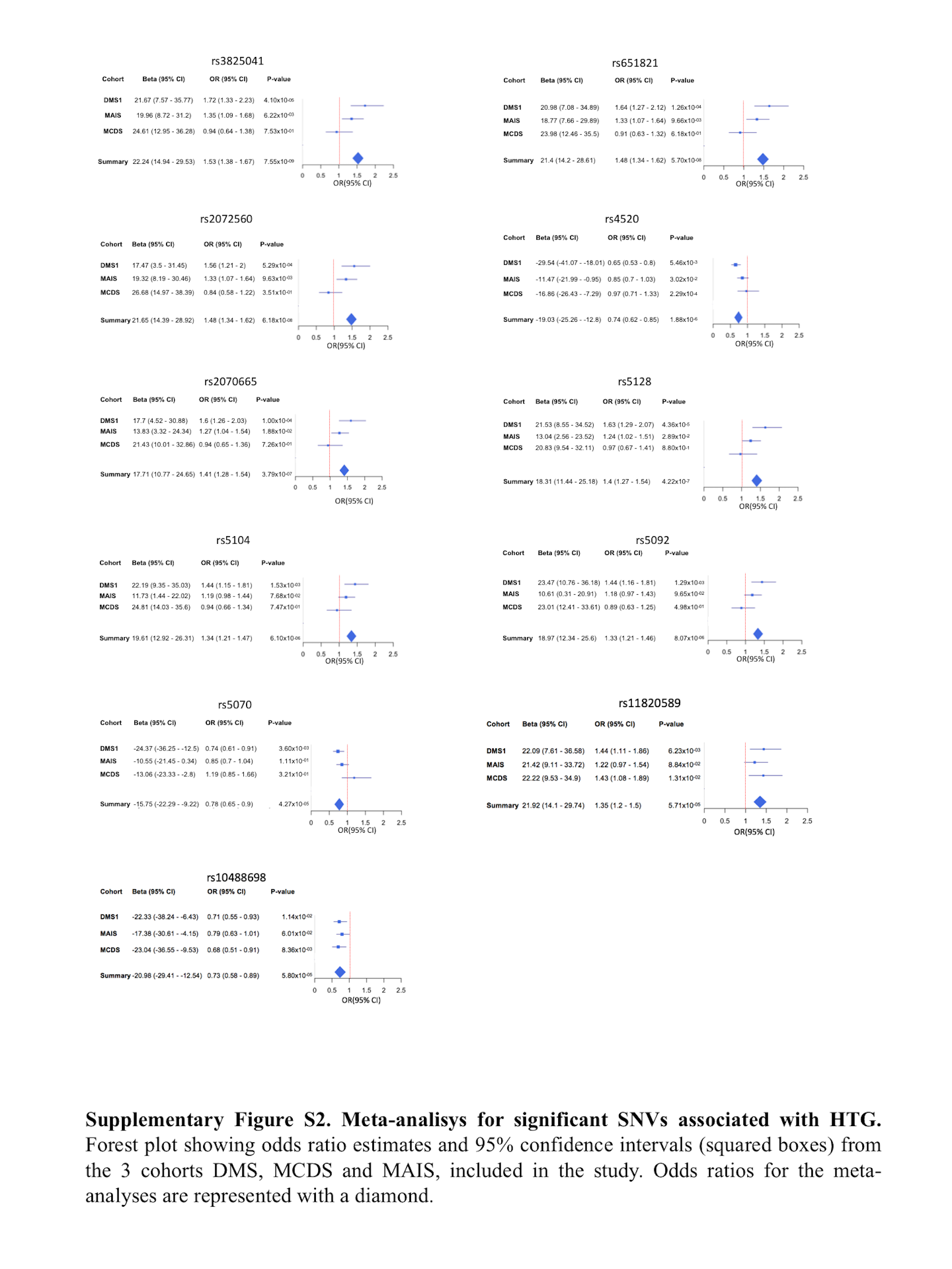

Supplement: Supplementary file 5 [file Image2.tiff]

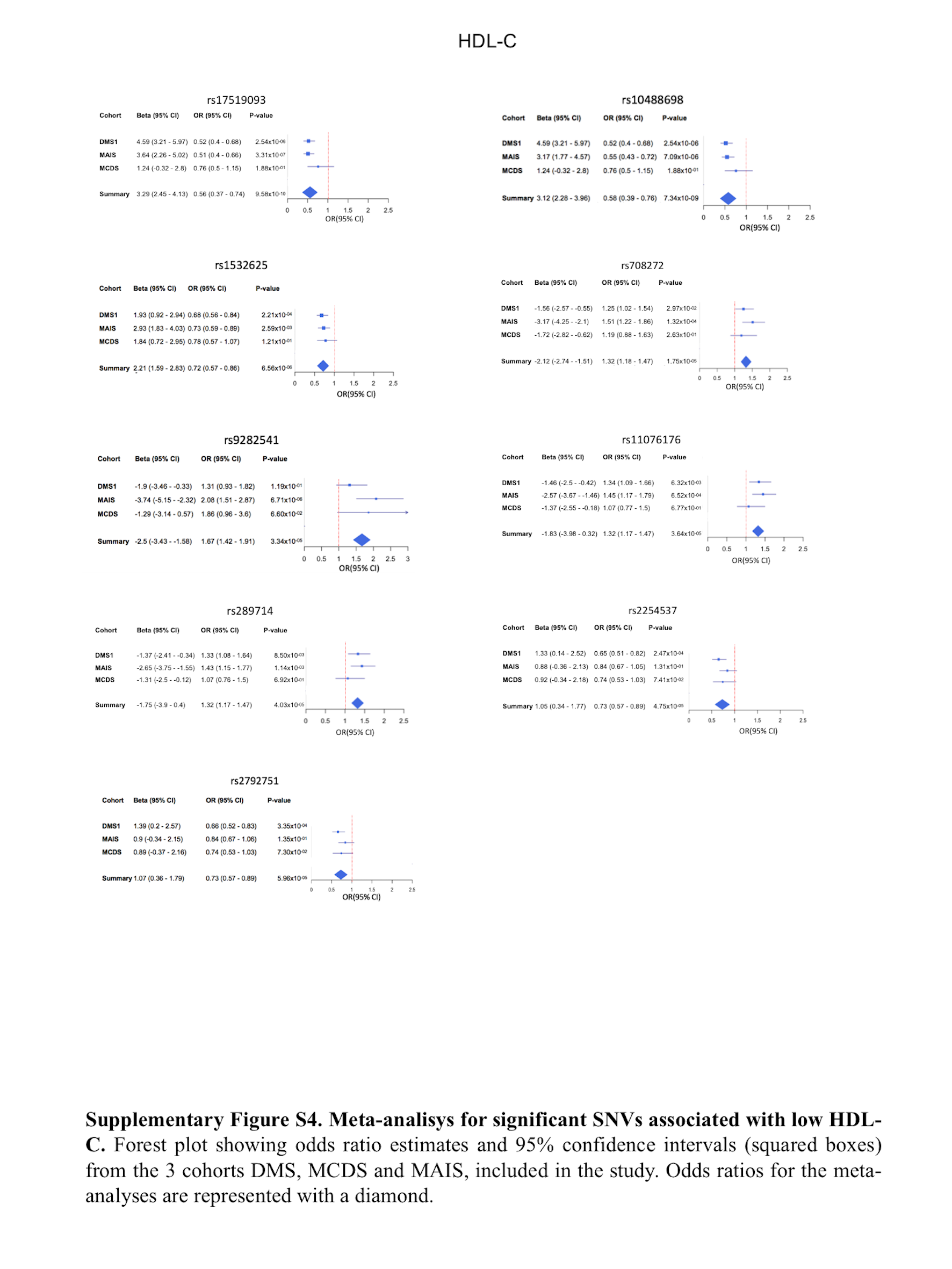

Supplement: Supplementary file 6 [file Image4.tiff]
